# TRPV1-Dependent Antiproliferative Activity of Dioecious *Maclura pomifera* Extracts in Estrogen Receptor-Positive Breast Cancer Cell Lines Involves Multiple Apoptotic Pathways

**DOI:** 10.3390/ijms25105258

**Published:** 2024-05-11

**Authors:** Mafia Mahabub Rumpa, Camelia Maier

**Affiliations:** Division of Biology, School of the Sciences, Texas Woman’s University, Denton, TX 76204, USA; mrumpa@twu.edu

**Keywords:** breast cancer, TRPV1, dioecious *Maclura pomifera*, ER-positive breast cancer, apoptosis, mitochondrial intrinsic apoptotic pathway, extrinsic apoptotic pathway, ER-dependent apoptosis

## Abstract

Globally, breast cancer is a significant cause of mortality. Recent research focused on identifying compounds regulating the transient receptor potential vanilloid 1 (TRPV1) ion channel activity for the possibility of developing cancer therapeutics. In this study, the antiproliferative properties and mechanisms of action through TRPV1 of *Maclura pomifera*, a dioecious tree native to the south-central USA, have been investigated. Male and female extracts of spring branch tissues and leaves (500 µg/mL) significantly reduced the viability of MCF-7 and T47D cells by 75–80%. *M. pomifera* extracts induced apoptosis by triggering intracellular calcium overload via TRPV1. Blocking TRPV1 with the capsazepine antagonist and pretreating cells with the BAPTA-AM chelator boosted cell viability, revealing that *M. pomifera* phytochemicals activate TRPV1. Both male and female *M. pomifera* extracts initiated apoptosis through multiple pathways, the mitochondrial, ERK-induced, and endoplasmic reticulum-stress-mediated apoptotic pathways, demonstrated by the expression of activated caspase 3, caspase 9, caspase 8, FADD, FAS, ATF4, and CHOP, the overexpression of phosphorylated PERK and ERK proteins, and the reduction of BCL-2 levels. In addition, AKT and pAKT protein expressions were reduced in female *M. pomifera*-treated cells, revealing that female plant extract also inhibits PI3K/Akt signaling pathways. These results suggest that phytochemicals in *M. pomifera* extracts could be promising for developing breast cancer therapeutics.

## 1. Introduction

Breast cancer has become a significant concern for women worldwide, as the number of reported cases reached a staggering 2.26 million in 2020, highlighting the need for more significant efforts in preventing, detecting, and treating this life-threatening disease. Breast cancer has now surpassed all other types of cancer as the leading cause of cancer-related deaths among women [1,2]. In the USA, estimated new cases increased by 31% in 2023 [3]. Although progress has been made in cancer diagnosis and treatment, the development of chemotherapeutic agents continues to be widely researched [4,5]. Plants have been used for medicinal purposes for thousands of years, and many plants contain chemical compounds that have shown potential in cancer treatment [6,7,8]. Various plant secondary metabolites, including alkaloids, flavonoids, terpenoids, and phenolics with anti-cancer properties could provide a broader range of therapeutic benefits [7,9,10].

There is a growing interest in creating new and less harmful cancer-fighting drugs from plants native to a given area that have been used in folk medicine [9,10,11]. *M. pomifera* (*Moraceae*), commonly known as Osage orange or hedge apple, is a dioecious (having separate male and female individuals) tree native to the south-central USA [12]. Several biological activities of *M. pomifera* have been reported, such as anti-inflammatory, antinociceptive, and antiproliferative [13,14,15,16,17]. Although *M. pomifera* has not been extensively studied for its anti-cancer properties, some research suggests that certain of its compounds, such as pomiferin, a prenylated isoflavone, have potential as anti-cancer agents [16]. The sexual dimorphism of *M. pomifera* biochemistry is not well documented. More research is needed to determine the mechanisms of antiproliferative activity of *M. pomifera* chemicals and evaluate their efficacy and safety, and especially determine the differences in antiproliferative activities and mechanisms of action of male and female extracts and chemicals.

Several chemicals derived from plants have been found to interact with transient receptor potential vanilloid 1 (TRPV1) channels and exhibit antiproliferative activity in different cancer cell lines [18,19]. TRPV channels are a part of the TRP channel superfamily, modulated by compounds of plant origin [20,21]. The TRPV1 channel is a non-selective cation channel primarily known for its role in pain and temperature sensation [22]. However, emerging evidence suggests that TRPV1 is also involved in various physiological and pathological processes, including cancer [18,23,24]. Capsaicin, gingerol, piperine, and resiniferatoxin are natural compounds derived from several plant species and well-known activators of the TRPV1 channel [21,24]. Studies have shown that these compounds can induce apoptosis in various cancer cell types, including breast cancer. The activation of TRPV1 causes calcium influx and triggers downstream signaling pathways that contribute to antiproliferative effects [18,25,26]. More research is required to fully comprehend the potential of phytochemicals targeting TRPV1 in cancer treatment.

The aim of the present investigation is to determine the antiproliferative mechanisms of action of male and female *M. pomifera* plant extracts on two estrogen receptor (ER)-positive breast cancer cell lines: MCF-7 with wild-type tumor suppressor protein p53 and T47D with mutated p53. We report that male and female *M. pomifera* plant extracts possess antiproliferative properties in ER-positive MCF-7 and T47D breast cancer cells without affecting the growth of human mammary epithelial cells (HMECs) and induce apoptosis through multiple pathways. The male plant extract induces apoptosis through four pathways and the female extract through five pathways. To our knowledge, this is the first study reporting the antiproliferative properties and mechanisms of action of male and female *M. pomifera* extracts in ER-positive MCF-7 and T47D breast cancer cells.

## 2. Results

### 2.1. Antiproliferative Activities of Male and Female M. pomifera in MCF-7 and T47D Cells

**Figure 1:**
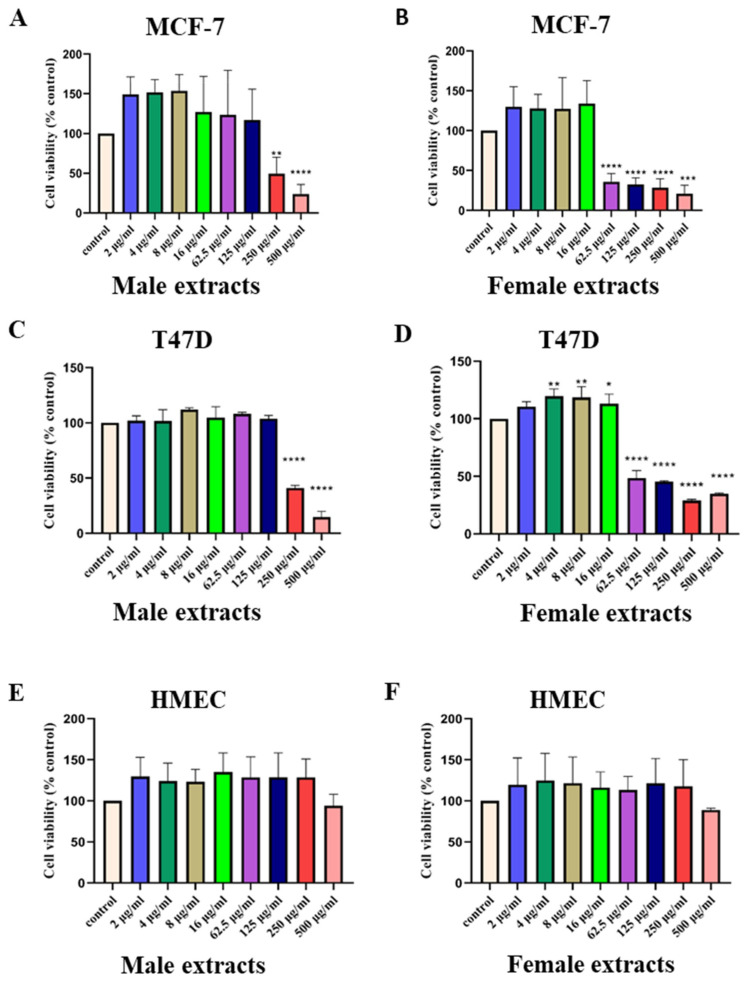
Antiproliferative activities of male and female *M. pomifera* extracts on ER-positive breast cancer cell lines: (**A**,**B**) MCF-7; (**C**,**D**) T47-D; and (**E**,**F**) effects of male and female *M. pomifera* extracts on the growth of HMECs. Values represent the mean ± standard deviation (SD) of three independent experiments. One-way ANOVA, followed by Tukey’s post hoc test, was performed. * *p* < 0.05, ** *p* < 0.01, *** *p* < 0.001, and **** *p* < 0.0001 vs. DMSO control set.

To test the antiproliferative activities of male and female *M. pomifera*, ER-positive MCF-7 and T47D cell lines were treated with increasing concentrations of *M. pomifera* male or female extracts. Both male and female *M. pomifera* extracts dose-dependently inhibited the proliferation of ER-positive MCF-7 (Figure 1A,B) and T47D cell lines (Figure 1C,D). The male extracts significantly inhibited cell viability starting at 250 µg/mL in both cell lines compared to the corresponding DMSO control cells. Significant cell viability inhibition started at 62.5 µg/mL extract concentration in female extract-treated MCF-7 and T47D cells. At 500 µg/mL, the male and female extracts significantly inhibited MCF-7 cell viability by 75% and 80%, respectively (Figure 1A,B), and in the T47D cell line, male extracts significantly inhibited cell viability by 80% (Figure 1C). Treatment with female extracts on T47D cells showed a significant biphasic effect. The proliferation of T47D cells was significantly increased at 4 µg/mL–16 µg/mL of *M. pomifera* female extract treatment, whereas extract concentrations of 62.5 µg/mL–500 µg/mL significantly inhibited proliferation (Figure 1C,D). *M. pomifera* male and female extracts did not inhibit the growth of HMECs (Figure 1E,F).

**Figure 2:**
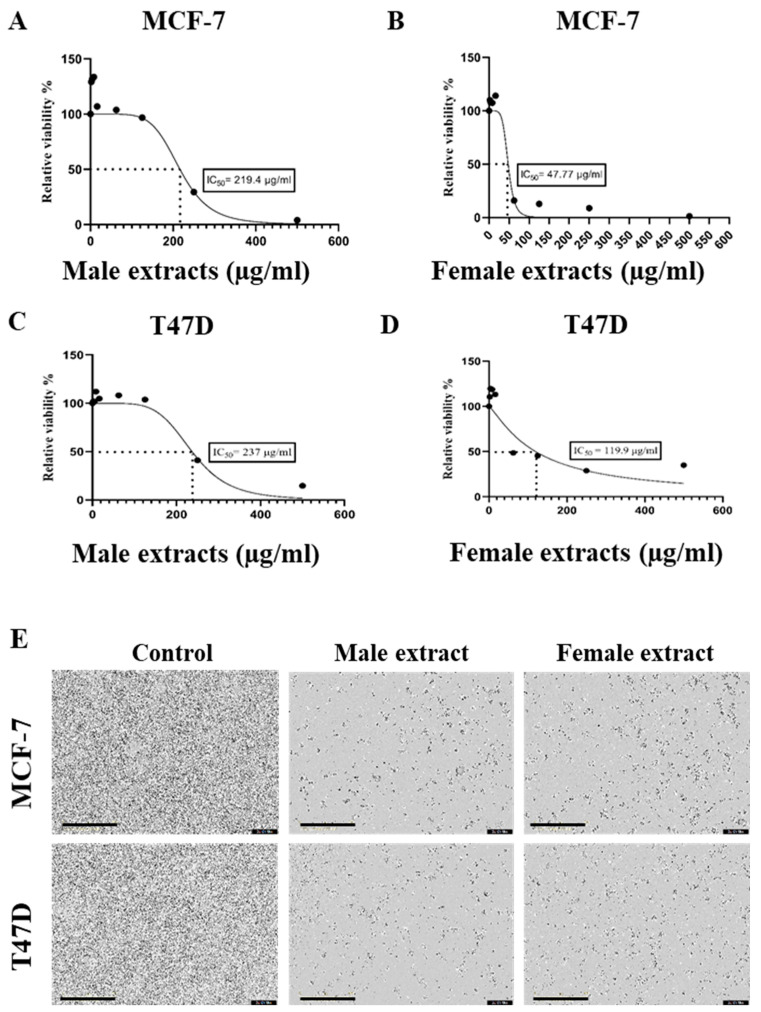
IC50 of male and female *M. pomifera* extracts on ER-positive breast cancer cell lines: (**A**,**B**) MCF-7 and (**C**,**D**) T47D. (**E**) Male and female *M. pomifera* treatment at 500 μg/mL resulted in morphological changes in MCF-7 and T47D cells. Cell morphological changes were observed for three days using the live cell imaging system IncuCyte, and images were taken on the third day after treatment. Scale bar: 400 μm.

The half-maximal inhibitory concentration (IC50) of male and female *M. pomifera* extracts was 219.4 µg/mL and 47.77 µg/mL, respectively, for MCF-7 cells (Figure 2A,B). The IC50 of male and female extracts against T47D cells was 237 µg/mL and 119.9 µg/mL, respectively (Figure 2C,D). Female extracts are more potent, having significantly lower IC50 values than male extracts (*p* < 0.05, *t*-test).

Cell morphological changes were observed after male and female *M. pomifera* extract treatment (500 μg/mL) in both cell lines. Three days after treatment, the *M. pomifera* extract treatments resulted in a noticeable cell loss, shrinkage, rounding, and partial and complete detachment from the bottom of the wells, confirming the cytotoxic effects of *M. pomifera* extracts on MCF-7 and T47D cells (Figure 2E).

### 2.2. Antiproliferative Activities of Osajin and Pomiferin in MCF-7 and T47D Cells

**Figure 3:**
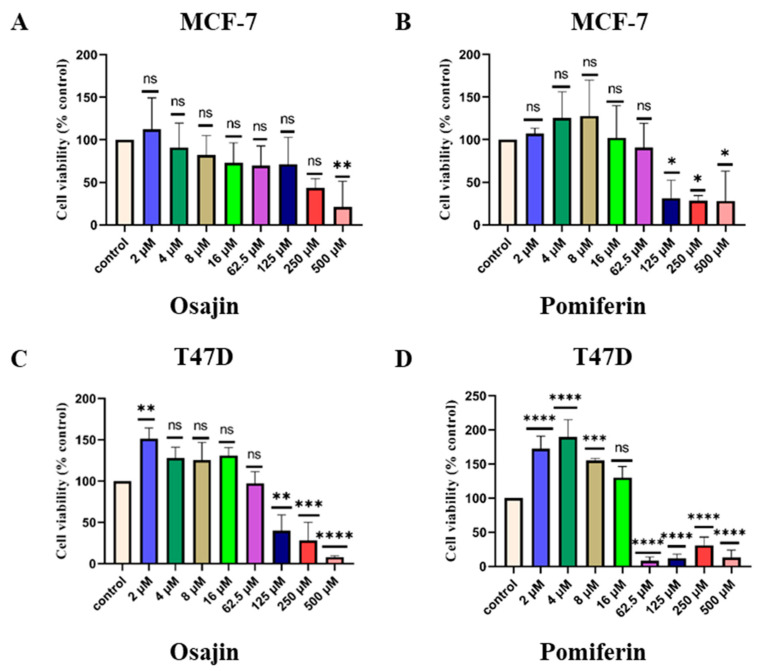
Antiproliferative activities of osajin and pomiferin on ER-positive breast cancer cell lines: (**A**,**B**) MCF-7 and (**C**,**D**) T47D. Values represent the mean *±* SD of three independent experiments. One-way ANOVA, followed by Tukey’s post hoc test, was performed. * *p* < 0.05, ** *p* < 0.01, *** *p* < 0.001, and **** *p* < 0.0001 vs. DMSO control set; ns = not significant.

Osajin and pomiferin are known *M. pomifera* phytochemicals [13]. Our preliminary chemical profiles of male and female plant extracts showed the presence of osajin (Table S1). To test the antiproliferative activities of osajin and pomiferin, ER-positive MCF-7 and T47D cell lines were treated with these phytochemicals at concentrations of 0.5 µM, 1 µM, 5 µM, 10 µM, 50 µM, 100 µM, 250 µM, and 500 µM. Osajin and pomiferin dose-dependently inhibited the proliferation of ER-positive MCF-7 cells. In the case of MCF-7 cells, at 500 µM, osajin inhibited cell viability by 75% (Figure 3A), while pomiferin started significantly inhibiting cell viability at 125 µM concentration, and at 500 µM, cell inhibition reached 75% (Figure 3B). For T47D cells, 125 µM osajin started significantly inhibiting cell viability, and at 500 µM, inhibition was 90% (Figure 3C). Pomiferin, at 62.5 µM concentration, started significantly inhibiting T47D cell viability by 95% (Figure 3D).

### 2.3. Male and Female M. pomifera Extracts Induce Apoptosis in MCF-7 and T47D Cells

**Figure 4:**
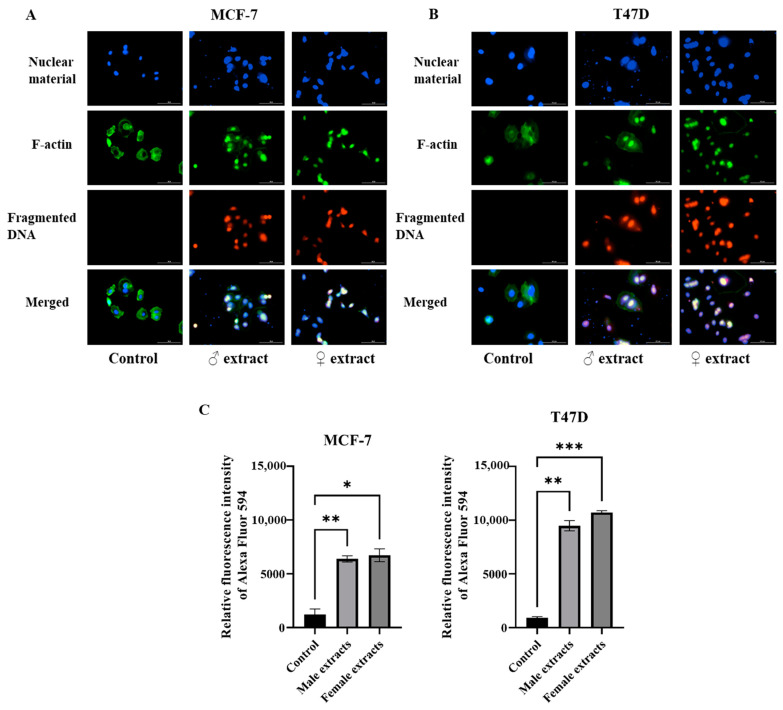
Apoptosis detected by means of TUNEL assay on MCF-7 (**A**) and T47D (**B**) cells treated with *M. pomifera* male or female extracts. Modified dUTP from the TUNEL assay kit binds to fragmented DNA of the apoptotic cells (red fluorescence). Hoechst 33342 and phalloidin were used to stain nuclear material (blue fluorescence) and F-actin (green fluorescence), respectively. Cells were visualized with LionHeart FX microscope; scale bar: 100 μm. (**C**) Relative fluorescence intensity of TUNEL assay. Red fluorescence (Alexa Fluor 594) was measured using LionheartTM FX Microscope BioTek Gen5.0 software. One-way ANOVA; * *p* < 0.05, ** *p* < 0.01, *** *p* < 0.001 vs. DMSO control set.

MCF-7 and T47D cells were treated with male or female *M. pomifera* extracts at 500 µg/mL for 20 h, and TUNEL assays were employed to detect apoptosis. Typical DNA fragmentation in both MCF-7 and T47D cell lines was observed compared to the DMSO control cells (Figure 4A,B). Male and female *M. pomifera* extract treatments significantly increased the numbers of apoptotic MCF-7 and T47D cells, shown by the relative fluorescence intensity of Alexa 594 (red fluorescence) (Figure 4C).

### 2.4. Blocking TRPV1 Activity Increased MCF-7 and T47D Cell Viability

**Figure 5:**
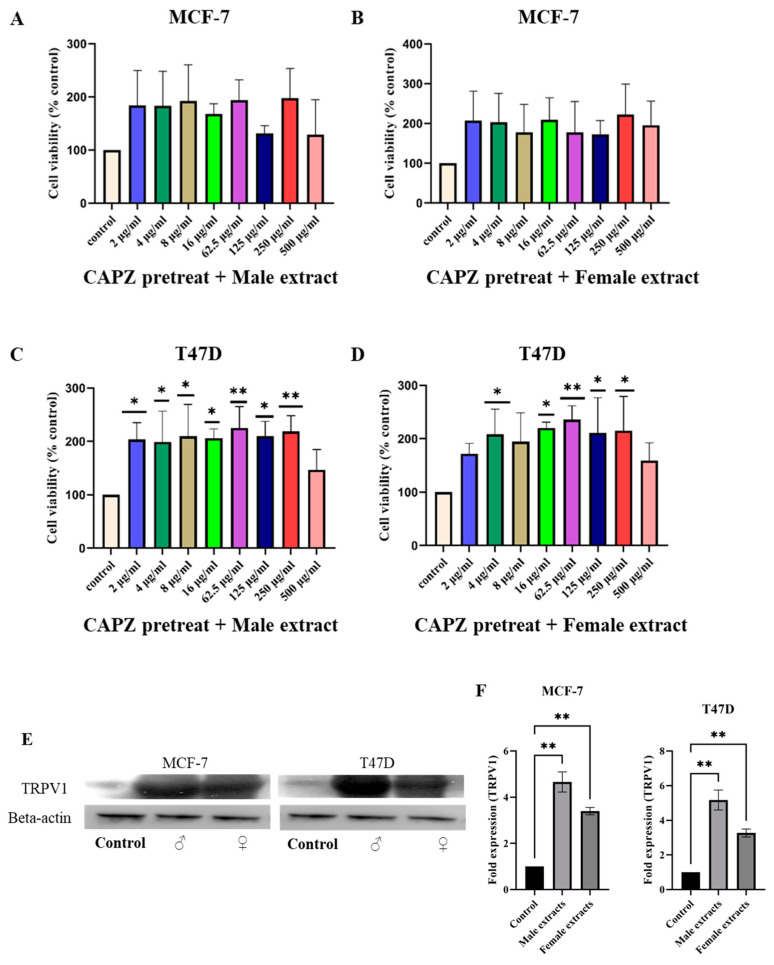
Blocking of TRPV1 increased cell viability of MCF-7 (**A**,**B**) and T47D (**C**,**D**) breast cancer cell lines. Cells were pretreated with 10 μM capsazepine (CAPZ), a TRPV1 antagonist, and then treated with male or female extracts. Cell viability was measured by means of an MTS assay. Values represent the mean ± SD of three independent experiments. One-way ANOVA, followed by Tukey’s post hoc test, was performed. * *p* < 0.05, ** *p* < 0.01 vs. DMSO control set. (**E**) Western blot and (**F**) relative protein expression of TRPV1 compared to untreated DMSO controls. MCF-7 and T47D cells were treated with 500 µg/mL concentrations of male and female *M. pomifera* for 17 h, and then cell lysates were isolated, and Western blot analyses of TRPV1 were performed. Beta-actin served as loading control. ImageJ software (https://imagej.net/ij/download.html accessed on 24 December 2023) was used to determine fold protein expression.

We hypothesized that *M. pomifera* extracts would activate TRPV1, and as a result, the calcium influx in the cells will contribute to apoptosis. To test the hypothesis, both MCF-7 and T47D cells were pretreated with 10 μM of capsazepine (CAPZ), a TRPV1 antagonist to block TRPV1 activity, and then were treated with male or female extracts. In the case of MCF-7 cells, TRPV1 inhibition increased cell proliferation. Capsazepine completely blocked the effect of both male and female *M. pomifera* extracts (Figure 5A,B). In the case of T47D cells, TRPV1 inhibition significantly increased the cell viability of treated cells compared to DMSO control cells, showing the blocking effect of capsazepine (Figure 5C,D). These results show that male and female *M. pomifera* extracts reduce the viability of MCF-7 and T47D cells in a TRPV1-dependent manner. Western blots were performed to check the TRPV1 protein expression in both cell lines. Immunoblot analyses showed significantly less expression of TRPV1 in DMSO control MCF-7 and T47D cells, suggesting that an activator is needed for TRPV1 protein expression. The TRPV1 protein was expressed in the male and female extract-treated MCF-7 and T47D cells (Figure 5E,F), revealing that both male and female plant extracts increased TRPV1 expression.

### 2.5. Calcium Chelation Increases MCF-7 and T47D Cell Viability

**Figure 6:**
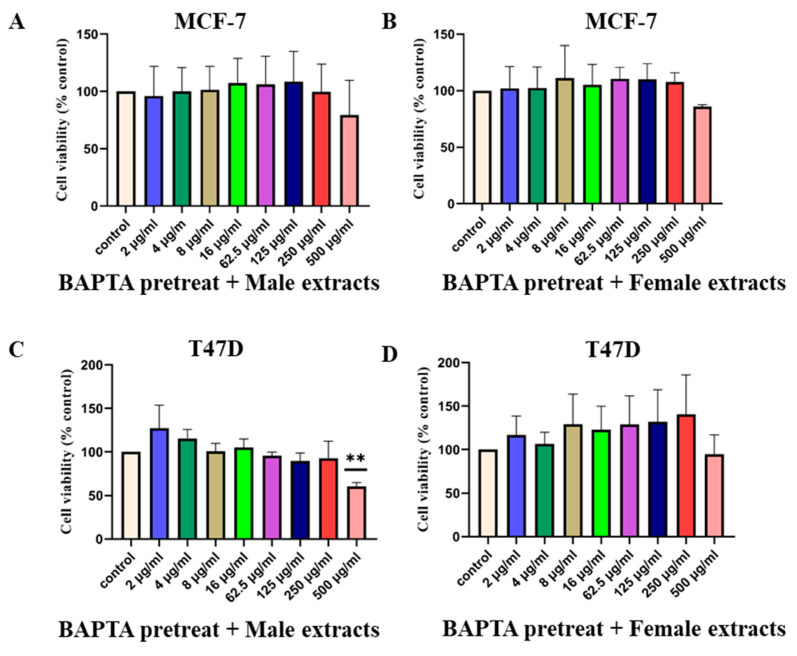
Calcium chelation blocks TRPV1 activity and increases cell viability of MCF-7 and T47D breast cancer cell lines: (**A**,**B**) MCF-7 and (**C**,**D**) T47-D. Cells were pretreated with 1 μM of BAPTA-AM, a calcium chelator, and then treated with male and female extracts. Cell viability was measured by means of an MTS assay. Values represent the mean ± SD of three independent experiments. One-way ANOVA, followed by Tukey’s post hoc test, was performed. ** *p* < 0.01 vs. DMSO control set.

The results presented so far show that *M. pomifera* extracts increase TRPV1 protein expression in MCF-7 and T47D cells, which resulted in a significant decrease in cell viability. TRPV1 activation allows calcium influx into cells, which triggers downstream signaling pathways, contributing to antiproliferative effects [25]. We hypothesized that chelating calcium ions may oppose the above scenario by blocking TRPV1 activity and increasing cell viability. MCF-7 and T47D cells were pretreated with 1 μM of the calcium chelator BAPTA-AM, and then treated with male or female extracts. In the case of MCF-7 cells, chelating calcium did not decrease cell viability. It completely blocked the effect of both male and female *M. pomifera* extracts (Figure 6A,B). In the case of female extract-treated T47D cells, chelating calcium did not decrease cell viability (Figure 6D). However, the 500 μg/mL male extract-treated T47D cells showed a significant reduction in cell viability, possibly revealing the cytotoxic effects of male plant extract at high concentrations (Figure 6C).

### 2.6. Male and Female M. pomifera Extracts Induce Mitochondrial Calcium Overload in MCF-7 and T47D Cells

**Figure 7:**
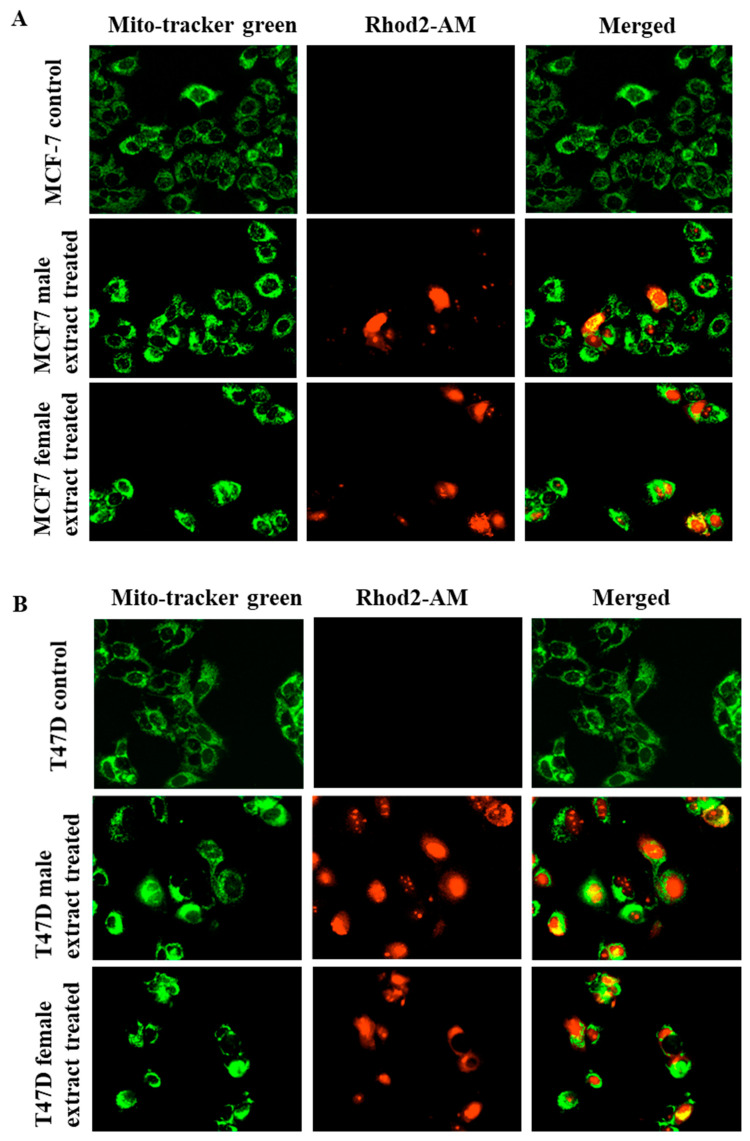
Male and female *M. pomifera* extracts induce mitochondrial calcium overload in MCF-7 (**A**) and T47D (**B**) cells. MCF-7 and T47D cells were simultaneously loaded with 10 μM of mito-Tracker Green and 5 μM of Rhod2-AM, and then exposed to male or female *M. pomifera* extracts. Within 15 s after adding the treatments, the corresponding fluorescence signal was monitored using a LionHeart FX microscope, 20× magnification.

Male and female *M. pomifera* extracts activate TRPV1, and therefore, calcium influx into cells is expected. We wanted to see if this activation can further trigger an overload of calcium in mitochondria, which contributes to the disruption of mitochondrial calcium homeostasis and apoptosis. Rhod2-AM, used to monitor mitochondrial calcium, was predominantly localized within the mitochondria of both MCF-7 and T47D cells, evidenced by the merged yellow staining pattern in Figure 7A,B.

### 2.7. Male and Female M. pomifera Extracts Induce Mitochondrial Intrinsic and Extrinsic Apoptotic Signaling Pathways in MCF-7 and T47D Cells

**Figure 8:**
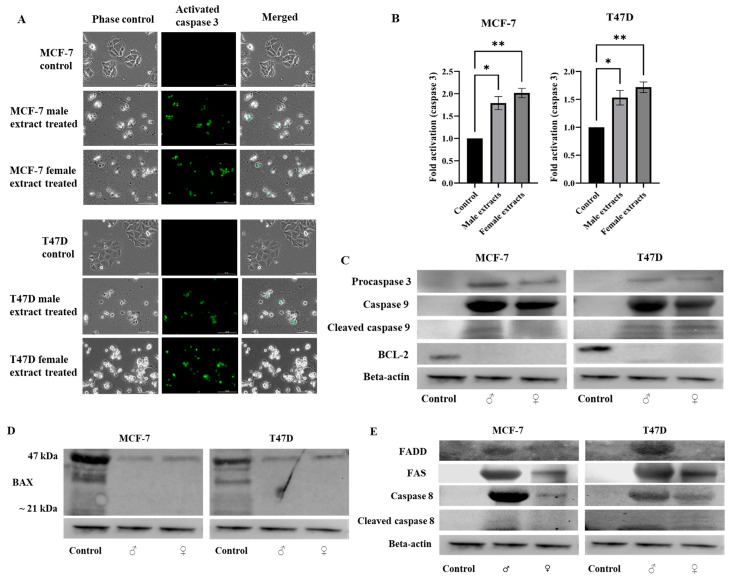
Male and female *M. pomifera* extracts induce mitochondrial intrinsic and extrinsic apoptosis in MCF-7 and T47D cells. (**A**) To check for cleaved caspase 3, MCF-7 and T47D cells were treated with 500 µg/mL male or female *M. pomifera* for 17 h, one drop of caspase 3/7 reagent was added, and the corresponding fluorescence signal was monitored using a LionHeart FX microscope, scale bar: 100 μm. (**B**) Fold activation of caspase 3 compared with DMSO control was measured using LionHeart BioTek Gen5.0 software. * *p* < 0.05, ** *p* < 0.01. (**C**,**D**) For intrinsic protein marker expressions, MCF-7 and T47D cells were treated with 500 µg/mL male or female *M. pomifera* for 17 h, cell lysates were isolated, and immunoblot analyses of procaspase 3, caspase 9, cleaved caspase 9, BCL-2, and BAX were performed. (**E**) Western blot analyses of mitochondrial extrinsic apoptotic proteins (FADD, FAS, caspase 8, and cleaved caspase 8). Representative Western blots, with beta-actin served as loading control.

Knowing that mitochondria play a vital role in the process of apoptosis, we further investigated the protein expression of apoptotic markers. Activated/cleaved caspase 3 was detected (green fluorescence) in male and female extract-treated MCF-7 and T47D cells (Figure 8A,B). Both *M. pomifera* male and female extract treatments led to the expression of procaspase 3, caspase 9, and cleaved caspase 9, an indication of mitochondrial intrinsic apoptosis, and the total eradication of the anti-apoptotic BCL-2 protein (Figure 8C). The proapoptotic BAX protein showed a higher molecular weight band (47 KDa) on Western blot (Figure 8D), a marker of apoptosis. The expression of FADD, FAS, and caspase 8, and the low expression of cleaved caspase 8 proteins were observed in the treated MCF-7 and T47D cells, indicating that *M. pomifera* treatments also led to extrinsic apoptosis (Figure 8E).

### 2.8. Male and Female M. pomifera Extracts Induce ERK-Dependent Apoptosis in MCF-7 and T47D Cells

**Figure 9:**
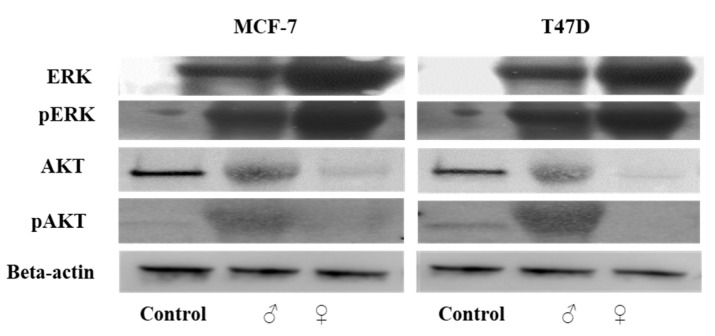
Male and female *M. pomifera* extracts induce ERK-dependent apoptosis in MCF-7 and T47D cells. Cells were treated with 500 µg/mL male or female *M. pomifera* extracts for 17 h, cell lysates were isolated, and immunoblot analyses of proteins AKT, pAKT, ERK, and pERK were performed. Representative Western blot, with beta-actin served as loading control.

The extracellular signal-regulated kinases (ERK1/2) and AKT proteins, also known as protein kinase B, regulate cellular signaling and play essential roles in cell survival, growth, and proliferation. A higher expression of phosphorylated ERK protein, known to mediate apoptosis, was detected in male and female extract-treated cells than in the untreated MCF-7 and T47D cells (Figure 9). The expression of phosphorylated and non-phosphorylated AKT proteins was higher in male extract-treated MCF-7 and T47D cell lines than in the female extract-treated cells. The reduced expression of AKT proteins in female extract-treated MCF-7 and T47D cell lines (Figure 9) suggests the inhibition of the PI3K/AKT signaling pathway by *M. pomifera* female extracts.

### 2.9. Male and Female M. pomifera Extracts Induce Endoplasmic Reticulum-Dependent Apoptosis in MCF-7 and T47D Cells

**Figure 10:**
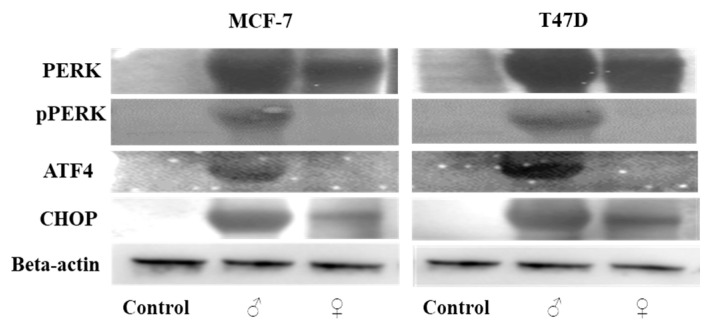
Male and female *M. pomifera* induce endoplasmic reticulum-dependent apoptosis in MCF-7 and T47D cells. MCF-7 and T47D cells were treated with 500 µg/mL male or female *M. pomifera* for 17 h, cell lysates were isolated, and Western blot analyses of endoplasmic reticulum-dependent apoptotic proteins PERK, pPERK, CHOP, and ATF4 were performed. Representative Western blot, with beta-actin served as the loading control.

To assess whether male and female *M. pomifera* extracts induce endoplasmic reticulum stress due to calcium overload, we further investigated the effect of both male and female *M. pomifera* on endoplasmic reticulum-dependent apoptotic proteins. MCF-7 and T47D cells were treated with 500 µg/mL male or female *M. pomifera* for 17 h, cell lysates were isolated, and Western blot analyses of endoplasmic reticulum-dependent apoptotic proteins were performed. Both male and female extract treatments led to a higher expression of protein kinase RNA-like ER kinase (PERK) and the C/EBP homologous protein (CHOP) (endoplasmic apoptotic protein). Phosphorylated PERK and activating transcription factor 4 (ATF4) proteins were detected only in male extract-treated MCF-7 and T47D cells (Figure 10).

## 3. Discussion

Breast cancer is the most often diagnosed illness globally [27], and therefore improvements in detecting and treating the disease must be expanded to reduce the global mortality rate of breast cancer. Natural substances with anti-cancer properties are present in many plants, and targeted research on bioactive plant chemicals could develop new breast cancer therapeutics [10]. The present study tested the antiproliferative activities of male and female *M. pomifera* on ER-positive MCF-7 (with wild-type tumor suppressor protein p53) and T47D (with mutant type p53) breast cancer cell lines and found that both male and female extracts significantly reduced the proliferation of both breast cancer cell lines (Figure 1, Figure 2 and Figure 3). It was previously reported that pomiferin isolated from *M. pomifera* fruit possesses antiproliferative activity on the MCF-7 tumorigenic breast epithelial cell line with low toxicity to non-tumorigenic breast epithelial cells (MCF-10A) [15]. A recent study revealed that *M. pomifera* leaf extract possesses anti-cancer activity on hepatic cancer (HepG2) cells but no specification of the plant sex from where the leaves were collected, and no mechanism of action were provided [28]. Zhao et al. (2013) showed that pomiferin isolated from *M. pomifera* fruit inhibits glioma stem-like cell (CD133+) growth and invasion [16]. Most studies on the antiproliferative effects of *M. pomifera* were performed with fruit extracts, or the sex of the plant was ignored when tissues were collected for extracts, as illustrated in the above-mentioned references. We conducted our study specifically with male and female plant extracts of *M. pomifera* based on the known sexual dimorphism in dioecious plants which could also involve biochemical dimorphism besides the morpho-anatomical characteristics and biological effects [29]; see Supplement 1. In our study, female extracts are more potent and have lower IC_50_ values than male extracts in their antiproliferative activity on MCF-7 and T47D breast cancer cell lines, suggestive of the biochemical composition dimorphism, which should be considered for dioecious plants, thus better serving the search for new therapeutics for breast and other types of cancer in the future.

A biphasic effect of the plant extracts on MCF-7 and T47D cell viability was observed. This could be explained by the agonistic or antagonistic effect of lower or higher concentrations of the *M. pomifera* plant extracts, respectively, on the different estrogen receptor subtypes present in the cells. Recent research showed that phytoestrogens, such as flavonoids and stilbenes, have a biphasic effect on cell proliferation, increasing it at low doses while inhibiting growth at high doses. Genistein and daidzein, known phytoestrogens, for example, promote the growth of ER-positive carcinomas at low concentrations and inhibit their growth by inducing apoptosis at high concentrations [30,31,32,33,34]. Therefore, in the case of ER-positive breast cancers, biphasic effects are observed due to interactions of phytoestrogens with ER-subtypes and the recruitment of cofactors that regulate gene expression and promote or prevent the progression of breast cancer cells [35]. The T47D cell line showed a significant increase in proliferation when treated with female *M. pomifera* extract and pomiferin, a phytoestrogen, compared to MCF-7 cells. This could be due to the mutant p53, known to inhibit apoptosis [36,37].

It has been shown that progesterone, through its receptor, increased levels of TRPV6 in T47D cell line [38]. Estrogen induces the expression of progesterone receptor [39]. Plants contain estrogen-like and progesterone-like compounds. Therefore, the interconnection between such phytochemicals, estrogen and progesterone receptors, and TRPV channel proteins in breast carcinoma should be further researched to find new therapeutics for breast cancer.

Osajin and pomiferin are two known prenylated phytoestrogens of *M. pomifera*. In our study, osajin and pomiferin showed antiproliferative activities on both MCF-7 and T47D cell lines in a similar pattern as for the *M. pomifera* plant extracts (Figure 1 and Figure 3). However, in MCF-7 cells, whole plant extracts significantly inhibited growth, starting at lowers concentrations than those of osajin and pomiferin, suggesting the presence of other phytochemicals besides osajin and pomiferin that contributed to the antiproliferative activity of the extracts. This finding is similar to the results obtained with male and female extracts, where the pattern of cell viability inhibition of osajin is similar to the effect of the male extract, and the pomiferin pattern is similar to that of the female extract, suggesting the biochemical sexual dimorphism of dioecious *M. pomifera*.

In our effort to identify the mechanism of action of *M. pomifera* extracts on decreasing cell viability on MCF-7 and T47D cells and knowing that high levels of intracellular calcium ions in breast cancer cells trigger apoptosis [40], we considered the effects of TRPV1, calcium influx, and apoptotic markers. Calcium ions are a common type of messenger in cells, and maintaining the balance of Ca^2+^ is crucial for regulating many cellular functions, including processes that are relevant to tumor growth and development, such as metabolism, apoptosis, and metastasis [40,41]. Zhai et al. (2020) reviewed TRPV1 effects in disrupting calcium homeostasis as potential target for the regulation of proliferation for treating cancer [25]. The TRPV superfamily of ion channels are one of the most active calcium-permeable channels across the plasma membrane regulating Ca^2+^ influx [18,26,42,43]. Experimental evidence showed that capsaicin can activate TRPV1 and showed potent anti-cancer activity against certain types of cancer both in Ca^2+^-dependent and -independent mechanisms [18,42]. In our study, both male and female *M. pomifera* extracts generated an increase in cytosolic Ca^2+^ through TRPV1 in MCF-7 and T47D cells, disrupting intracellular calcium homeostasis and inducing apoptosis. Blocking TRPV1 activity with capsazepine and calcium chelation increased cell viability, suggesting that the activation of TRPV1 by male and female *M. pomifera* extracts is the underlying mechanism of cell death in MCF-7 and T47D cells. Our results illustrated that *M. pomifera* extracts activated TRPV1, overloaded cells with calcium, and ultimately induced apoptosis in MCF-7 and T47D cells. Therefore, one approach to novel drug targets for breast cancer is to administer a channel activator to induce calcium influx that could trigger cell death.

*M. pomifera* extracts not only that activated TRPV1 influx of Ca^2+^, but they also significantly increased TRPV1 expression in MCF-7 and T47D cells (Figure 5E,F), resulting in apoptosis. Increasing TRPV1 expression blocks mitosis and induces apoptosis in different cancer cell lines [44]. It has been shown that capsaicin induces high TRPV1 expression and apoptosis in breast cancer cell lines [45,46,47]. *M. pomifera* extracts inhibited the viability of both MCF-7 and T47D cells, whereas no inhibition of the viability of normal HMECs was observed. It seems that extract chemicals have high selectivity towards cancer cells. The differences between the mechanisms of action of plant extract treatments on the cancer vs. normal cells are not well understood. Normal cells have low to no expression of TRPV1 compared to cancer cells [44]; therefore, they are less susceptible and sensitive to calcium changes induced by the activation of TRPV1 channels. Cancer cells, on the other hand, are highly susceptible and sensitive to calcium changes, which may be linked to the overexpression of Ca^2+^-related genes and inability to maintain homeostasis.

**Figure 11:**
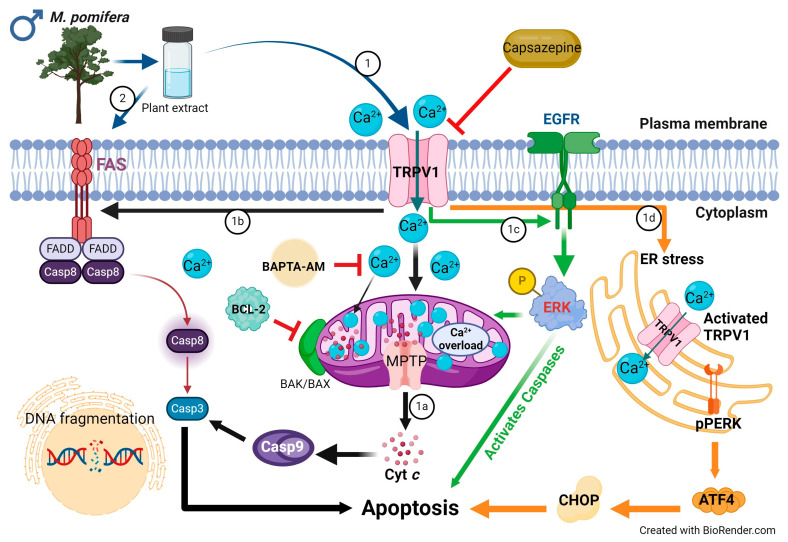
Proposed model of the mechanisms of action of male *M. pomifera* extracts on ER-positive MCF-7 and T47D breast cancer cells. (1) Plant extract activates TRPV1, inducing calcium influx into cells. The elevated intracellular calcium triggers several apoptotic pathways, the mitochondria-mediated intrinsic pathway, extrinsic FAS-FADD and ERK-induced pathways, and ER-stress mediated pathway. (1a) The accumulation of Ca^2+^ into mitochondria causes the transient depolarization of mitochondrial membrane potential. As a result, the mitochondrial permeability transition pores (MPTPs) open and release cytochrome c (cyt c), leading to caspase activation, increasing proapoptotic proteins BAK/BAX, decreasing anti-apoptotic BCL-2, and leading to apoptosis. (1b) Calcium influx triggers the extrinsic pathway by activating FAS, FADD, caspase 8, and caspase 3, and leading to apoptosis. It may be that plant extracts activate the FAS/FADD pathway directly (2). (1c) TRPV1 also activates the epidermal growth factor receptor (EGFR), further activating ERK. Phosphorylated ERK then activates caspases by inducing mitochondrial intrinsic apoptosis. (1d) Plasma membrane TRPV1 activation and calcium influx lead to endoplasmic reticulum (ER) stress. ER TRPV1 becomes another source of calcium release in the cell. ER stress activates PERK, phosphorylated PERK (pPERK) activates ATF4, and ATF4 activates CHOP, leading to PERK-ATF4-CHOP-mediated apoptosis.

**Figure 12:**
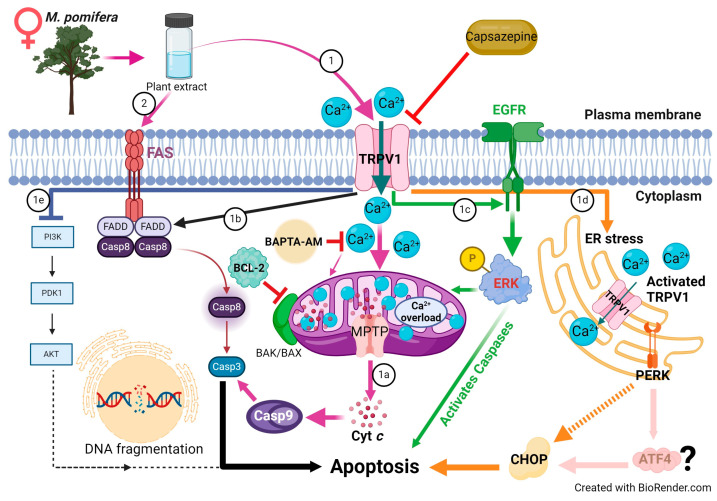
Proposed model of the mechanisms of action of female *M. pomifera* on ER-positive MCF-7 and T47D breast cancer cells. Similar to the mechanisms of action of male plant extract, *M. pomifera* female extract also (1) activates TRPV1, and initiates (1a) mitochondrial intrinsic, (1b) extrinsic, (1c) ERK-dependent, and (1d) ER-stress-mediated apoptosis. (1e) In addition, female *M. pomifera* extract downregulates the PI3K-AKT signaling pathway. It may be that plant extracts activate the FAS/FADD pathway directly (2).

Studies have revealed that the disruption of calcium homeostasis collapses mitochondrial membrane potential, reduces ATP production, increases ROS levels, and releases proapoptotic proteins [24,48]. In our study, mitochondrial Ca^2+^ overload was observed after *M. pomifera* extract treatments in both MCF-7 and T47D cells (Figure 8). To investigate the molecular mechanisms underlying TRPV1-dependent *M. pomifera*-induced cell death, we checked the expression of apoptotic protein markers on the mitochondrial intrinsic and extrinsic apoptotic pathways. Mitochondrial depolarization induces cytochrome c release into the cytoplasm, activating caspase-3 via caspase-9 by interacting with Apaf-1 [49]. Activated caspase-3 is one of the hallmarks of apoptosis induced by several natural products [10,50]. *M. pomifera* extracts induced downstream caspase-3/9 activity, highlighting the induction of the apoptosis program, as presented in the proposed models of apoptotic mechanisms in Figure 11 and Figure 12. Although T47D cells have mutated p53 tumor suppressor, it seems that chemicals in plant extracts activated other apoptotic pathways than the p53 dependent apoptotic pathway.

Anti-apoptotic BCL-2 protein protects against cell death by apoptosis and allows senescent cells to survive [49]. We observed no expression of anti-apoptotic BCL-2 protein in either male or female extract-treated MCF-7 and T47D cells, suggesting that *M. pomifera* could be an excellent candidate to target BCL-2 proteins in different cancers. Our results show that the BAX protein is expressed in high molecular weight. A study revealed that in apoptotic cells, BAX oligomerizes, which is required for its proapoptotic activity, appears as higher molecular-weight bands in Western blots, and binds to the mitochondrial membrane inducing mitochondrial apoptosis [51].

*M. pomifera* extracts activated the extrinsic apoptotic pathway as observed in the expression of FAS, FADD, and caspase 8 proteins (Figure 11 and Figure 12). Previous studies, which corroborate our results, reported that the TRPV1 N-terminus can bind to proapoptotic FAS-associated proteins and activate extrinsic apoptotic pathways [52,53,54]. A faded FADD Western blot band in the case of female extract-treated MCF-7 and T47D cells (Figure 8) may indicate that female *M. pomifera* extract treatments activate other FAS-associated death ligands than FADD.

It has been shown that TRPV1 activates external growth factor receptor (EGFR) and its related protein signaling pathway and upregulates ERK1/2 activation [55]. Phosphorylated ERK protein can suppress cell growth and induce mitochondrial apoptotic proteins [56]. In our study, we also found phosphorylated ERK (pERK) protein activation in both male and female *M. pomifera* extract-treated MCF-7 and T47D cells (Figure 11 and Figure 12).

TRPV1 is also found in the endoplasmic reticulum membrane [57] and its activation causes ER stress [58], thus serving as another source of calcium release in the cell. Endoplasmic reticulum-mediated stress and the compromised mitochondria will subsequently release active signals to carry out apoptosis [18,48]. Our results show that male and female *M. pomifera* extracts induce endoplasmic reticulum stress, and ultimately induce apoptosis in MCF-7 and T47D breast carcinoma cells. TRPV1 activates PERK, phosphorylated PERK phosphorylates eIF2 and, as a result, reduces global protein translation while increasing ATF4 translation. ATF4 stimulates the expression of CHOP, a transcription factor that increases the expression of other apoptotic factors [59] (Figure 11 and Figure 12). Our results show a high expression of pPERK, ATF4, and CHOP proteins in the male *M. pomifera* extract-treated cells. However, in the female extract-treated cells, no pPERK or ATF4 expression was found, suggesting that other factors may be activating CHOP in female extract-treated MCF-7 and T47D cells.

Our results suggest that female *M. pomifera* extract is involved in targeting the PI3K/AKT signal transduction pathway, precisely downregulating it (Figure 12). The serine/threonine kinase AKT, also known as protein kinase B (PKB), has numerous functions in the regulation of various cellular processes, such as cell survival, growth, proliferation, cell cycle, and metabolism. The balance between the loss and gain of AKT activation underlies the pathophysiological properties of cancer [60]. We observed a higher expression of total and phosphorylated AKT protein in male than female extract-treated cells (Figure 9). Studies have shown that the activation of the PI3K/AKT pathway contributes to tumorigenesis, and the inhibition of AKT can result in both decreased cellular proliferation and increased cell death [61,62]. TRPV1 can contribute to cell proliferation by activating serine-threonine kinase AKT [25]. However, agonists like capsaicin and resiniferatoxin and capsazepine antagonist can inhibit cell growth, but no mechanism of action has been described [63]. Therefore, our results suggest that female *M. pomifera* extract could become a potential candidate for targeting PI3K/AKT signaling pathways of breast cancer.

## 4. Materials and Methods

### 4.1. Plant Collection and Preparation of Extracts

Male and female *M. pomifera* stem and leaf tissues (young branches) were collected from Denton, TX, USA, during spring (March and April). Plant tissues (50 g) were extracted in 95% ethanol (1:4 *w*/*v*) at room temperature for two days and centrifuged at 2500× *g* for 20 min. Supernatants were filtered through Whatman filter paper #54 (Thomas Scientific, Swedesboro, NJ, USA). The filtrates were transferred to a pre-weighted vial and evaporated to dryness under nitrogen gas flow [64]. One gram was dissolved in 1 mL of dimethyl sulfoxide (DMSO) and stored at −20 °C until use.

### 4.2. Cell Lines and Cell Culture Conditions

ER-positive MCF-7 and T47D breast cancer cell lines and adult HMECs were obtained from American Type Culture Collection (ATCC, Manassas, VA, USA). MCF-7 breast carcinomas were maintained in Dulbecco’s Modified Eagle’s Medium (DMEM; ThermoFisher Scientific, Waltham, MA, USA), supplemented with 10% fetal bovine serum and 1% penicillin and streptomycin. T47D cells were maintained in RPMI-1640 medium, containing 10% fetal bovine serum and 1% penicillin and streptomycin (ThermoFisher Scientific). All cell lines were maintained in the logarithmic growth phase through routine passage every 2–3 days using 0.05% trypsin-EDTA treatment (ThermoFisher Scientific). After 75–80% confluence, the cells were trypsinized and transferred to a glucose-containing DMEM without glutamine and phenol-red (ThermoFisher Scientific). Cells were maintained in a phenol-red-free medium until they reached 75–80% confluence and then seeded for further experiments. Adult HMECs were grown in mammary epithelial basal medium (ATCC) supplemented with mammary epithelial cell growth kit (ATCC). All cell lines were incubated under a humid atmosphere of 5% (*v*/*v*) CO_2_ at 37 °C.

### 4.3. Cell Culture Treatments

All cells were seeded into 96-well cell culture plates at 10,000 cells/well and incubated for 24 h at 37 °C. After 24 h, the cells were treated with different concentrations of male and female *M. pomifera* extracts in DMSO (2, 4, 8, 16, 62.5, 125, 250, and 500 µg/mL), and osajin and pomiferin at 0.5, 1, 5, 10, 50, 100, 250, and 500 µM concentrations. Adult HMECs were treated with the above-mentioned concentrations of *M. pomifera* extracts. The final concentration of DMSO was <0.1%.

### 4.4. Antiproliferative Assays

Antiproliferative activity was evaluated by performing an MTS [3-(4,5-dimethylthiazol-2-yl)-5-(3-carboxymethoxyphenyl)-2-(4-sulfophenyl)-2H-tetrazolium] assay (Abcam, Boston, MA, USA). Briefly, cells were seeded into a 96-well plate (10,000 cells/well) and exposed to various concentrations of male and female *M. pomifera* extracts, osajin, or pomiferin. After 72 h of treatments, 10 µL of MTS reagent was added to each well and incubated at 37 °C for 2.5 h. The absorbance was measured at 490 nm using a Biotek’s Synergy HT plate reader. Three separate experiments, each containing three replications, were used to conduct the antiproliferative assays.

### 4.5. IC50 Estimation

The MTS assay results were used to calculate IC50 using the GraphPad Prism 9.4 software (GraphPad, La Jolla, CA, USA). A dose–response curve was fitted using nonlinear regression.

### 4.6. Visualization of Cytotoxic Effects

The morphological changes of the cells were observed using the live cell imaging system IncuCyte S3 (Sartorius, Ann Arbor, MI, USA) for several days. The cells were seeded into a 96-well plates (10,000 cells/well), exposed to 500 µg/mL of male and female *M. pomifera* extracts, and placed into the IncuCyte S3 live cell imaging system. The system was programmed to capture high-resolution bright field photos every 4 h.

### 4.7. Detection of Apoptosis

Cell apoptosis was observed by employing the Click-iT™ Plus TUNEL (the terminal deoxynucleotidyl transferase-mediated dUTP nick end labeling) assay kit (ThermoFisher Scientific). Cells were grown on coverslips inside a 6-well plate and treated with male and female *M. pomifera* extracts at 500 µg/mL concentrations. Twenty hours after treatment, the MCF-7 and T47D cells were fixed with 4% formaldehyde. TUNEL assays were performed according to the manufacturer’s instructions; the nuclear fluorescence of cells was detected and analyzed using a LionHeart FX microscope (Agilent, Santa Clara, CA, USA).

### 4.8. Capsazepine Treatments to Block TRPV1

Cells were seeded into 96-well cell culture plates at 10,000 cells/well and incubated for 24 h at 37 °C. After 24 h, the cells were pretreated with 10 µM of capsazepine (Abcam), a TRPV1 antagonist, for 30 min, and then treated with male or female *M. pomifera* extracts. The plates were incubated for 72 h at 37 °C. Cell viability was measured using MTS assays.

### 4.9. Calcium Chelation

Cells were seeded into 96-well cell culture plates at 10,000 cells/well and incubated for 24 h at 37 °C. After 24 h, the cells were pretreated with 1 µM of BAPTA-AM (Abcam), calcium chelator, for 30 min, and then treated with male and female *M. pomifera* extracts. The plates were incubated for 72 h at 37 °C. Then, cell viability was measured using MTS assays.

### 4.10. Visualization of Mitochondrial Calcium

Mitochondrial calcium was measured using Rhod2-AM (Abcam). MCF-7 and T47D cells were grown in 6-well plates and simultaneously loaded with 5 μM of Rhod2-AM and 10 μM of Mito-Tracker Green (ThermoFisher Scientific), and then exposed to male and female *M. pomifera* extracts. Within 15 s, the corresponding fluorescence signals were monitored with a LionHeart FX microscope in red fluorescence and green fluorescence channels.

### 4.11. Caspase 3 Activation Assay

All cells were seeded into 6-well cell culture plates at 2 × 10^5^ cells/well and incubated for 24 h at 37 °C. After 24 h, the cells were treated with male and female *M. pomifera* extracts. The plates were incubated at 37 °C for 17 h. One drop of the CellEvent™ Caspase-3/7 Green ReadyProbes™ reagent (ThermoFisher Scientific) was added in each well. After a 30 min incubation, cell fluorescence was detected and analyzed with a LionHeart FX microscope.

### 4.12. Western Blotting

Western blotting was carried out, as previously reported by Xu et al. (2020), with a few changes [18]. Cellular total protein was used to analyze the proapoptotic and anti-apoptotic proteins. The cell lysates were prepared using RIPA lysis and extraction buffer (ThermoFisher Scientific) supplemented with Halt™ protease and phosphatase inhibitor cocktail (ThermoFisher Scientific) and centrifuged at 12,000× *g* at 4 °C for 5 min. The supernatant was collected, and the total protein concentrations were estimated using a Pierce™ 660 nm Protein Assay Kit (ThermoFisher Scientific). Protein samples were subjected to 12% sodium dodecylsulfate polyacrylamide gel electrophoresis (SDS-PAGE) and were transferred onto polyvinylidene difluoride (PVDF) membranes (Bio-Rad Laboratories, Hercules, CA, USA). The membranes were incubated in blocking buffer [0.1% (*v*/*v*) Tween 20 in Tris-buffered saline, pH 7.4, with 5% (*w*/*v*) skim milk] at room temperature for 2 h and then probed with antibodies in a blocking buffer at 4 °C overnight. The antibodies used were ordered from Santa Cruz Biotechnology (Dallas, TX, USA): anti-beta actin (mouse monoclonal antibody conjugated with horseradish peroxidase (HRP), anti-caspase 3 (mouse monoclonal antibody conjugated with HRP; 1:1000, *v*/*v*), anti-caspase 9 (mouse monoclonal antibody conjugated with HRP; 1:1000, *v*/*v*), anti-caspase 8 (mouse monoclonal antibody conjugated with HRP; 1:1000, *v*/*v*), anti-TRPV1 (mouse monoclonal antibody conjugated with HRP; 1:1000, *v*/*v*), anti-CHOP (mouse monoclonal antibody conjugated with HRP; 1:1000, *v*/*v*), anti-FAS (mouse monoclonal antibody conjugated with HRP; 1:1000, *v*/*v*), anti-FADD (mouse monoclonal antibody conjugated with HRP; 1:1000, *v*/*v*), anti-PERK (mouse monoclonal antibody conjugated with HRP; 1:1000, *v*/*v*), anti-ERK (mouse monoclonal antibody conjugated with HRP; 1:1000, *v*/*v*), anti-pERK (mouse monoclonal antibody conjugated with HRP; 1:1000, *v*/*v*), anti-AKT (mouse monoclonal antibody conjugated with Alexa 488; 1:1000, *v*/*v*), and anti-pAKT (mouse monoclonal antibody conjugated with Alexa 488; 1:1000, *v*/*v*). The membranes were washed three times with Tris-buffered saline containing 0.1% (*v*/*v*) Tween 20 (TBST) for 5 min each, incubated with enhanced chemiluminescence substrate solution (Bio-Rad Laboratories) for 5 min, according to the manufacturer’s instructions, and visualized with a ChemiDoc system. For anti-BCL-2 (Abcam), anti-BAX (Abcam), and anti-pPERK (rabbit monoclonal antibody; 1:1000, *v*/*v*) (Cell Signaling Technology, Danvers, MA, USA), after overnight incubation, the membranes were washed three times with Tris-buffered saline containing 0.1% (*v*/*v*) Tween 20 (TBST) for 5 min each, after which they were incubated with secondary antibody [Goat Anti-Rabbit IgG H&L (Alexa Fluor^®^ 488); 1:500 *v*/*v*] for 1 h. After incubation, the membranes were washed three times with TBST buffer and immediately visualized using a ChemiDoc system (Bio-Rad Laboratories).

### 4.13. Statistical Analyses

Means and standard errors of the mean were calculated. One-way ANOVA, followed by Tukey’s post hoc test, was performed to determine the significant differences among the means for the antiproliferative assays using GraphPad Prism 9.4. A *p*-value < 0.05 was considered statistically significant. Half maximal inhibitory concentration (IC50) values were calculated by means of nonlinear regression analysis, followed by a paired Student *t*-test. ImageJ software (NIH, https://imagej.net/ij/download.html, accessed on 24 December 2023) was used to quantify Western blot bands.

## 5. Conclusions

Our study presents, for the first time, the TRPV1-dependent antiproliferative activity of male and female *M. pomifera* and their mechanisms of action in ER-positive MCF-7 and T47D cells. Both male and female *M. pomifera* plant extracts activate TRPV1 and induce mitochondrial intrinsic, extrinsic, and endoplasmic reticulum stress-mediated apoptotic pathways. In addition, the female *M. pomifera* plant extract downregulates the PI3K/AKT signaling pathway. *M. pomifera* extracts not only activate TRPV1 channel proteins but also increase their expression. The balance between the expression of TRPV1 and the triggered calcium influx into the cells are important factors controlling breast cancer cell proliferation. Our findings suggest that *M. pomifera* could become a potential source for designing cancer cell type-specific therapeutics.

## Data Availability

Data are contained within the article or Supplementary Materials.

## Supplementary Materials

The following supporting information can be downloaded at: https://www.mdpi.com/article/10.3390/ijms25105258/s1.
