# Supplementary material for: TRPV1-Dependent Antiproliferative Activity of Dioecious Maclura pomifera Extracts in Estrogen Receptor-Positive Breast Cancer Cell Lines Involves Multiple Apoptotic Pathways
Source: Int J Mol Sci. 2024 May 11;25(10):5258. doi: 10.3390/ijms25105258 (PMC11120667; doi:10.3390/ijms25105258)
Supplement: Supplementary file 1 [file ijms-25-05258-s001.zip › ijms-2905979-supplementary.pdf]

Preliminary Chemical Profile of M. pomifera Extracts

Same amounts of male and female *M. pomifera* plant material (50 g) were extracted with hexane in a ratio of 1:4 w/v. Extracts were evaporated to dryness re-suspended in ethanol (95%). Analyses of chemicals and compound confirmation were carried out by using ultra performance liquid chromatography electrospray ionization tandem mass spectrometry (UPLC-ESI-MS/MS) in a positive ionization mode as previously described (Basu et al., 2019). Stock solutions of standard compounds were prepared in ethanol and used for compound confirmation by direct comparison of mass spectra, characteristic fragmentation, and characteristic retention times. Each extract was analyzed in triplicate. Percentages of the identified phytochemicals were determined utilizing an external calibration method (diethylphenylmalonate) (Table S1).

Table S1. Relative concentration (%) of identified phytochemicals in *M. pomifera* extracts.

Phytochemical	Male *M. pomifera* extract (%)	Female *M. pomifera* extract (%)
Chalcone	0.14	0.13
Daidzein	0.052	0.015
Coumestrol	0.004	0.002
Genistein	0.0001	0.0002
Naringenin	0.0027	0.0004
Biochanin A	0.15	0.28
Kaempferol	0.003	0.002
Abietic acid	0.002	0.004
Rutin	0.0001	0.0007
Osajin	0.003	0.002

Basu, P.; Tongkhuya, S. A.; Harris, T. L.; Riley, A. R.; Maier, C.; Granger, J.; Wojtaszek, J.; Averitt, D. L. *Euphorbia bicolor* (*Euphorbiaceae*) latex phytochemicals induce long-lasting non-opioid peripheral analgesia in a rat model of inflammatory pain. *Front. pharmacol.* **2019**, 10, 958.

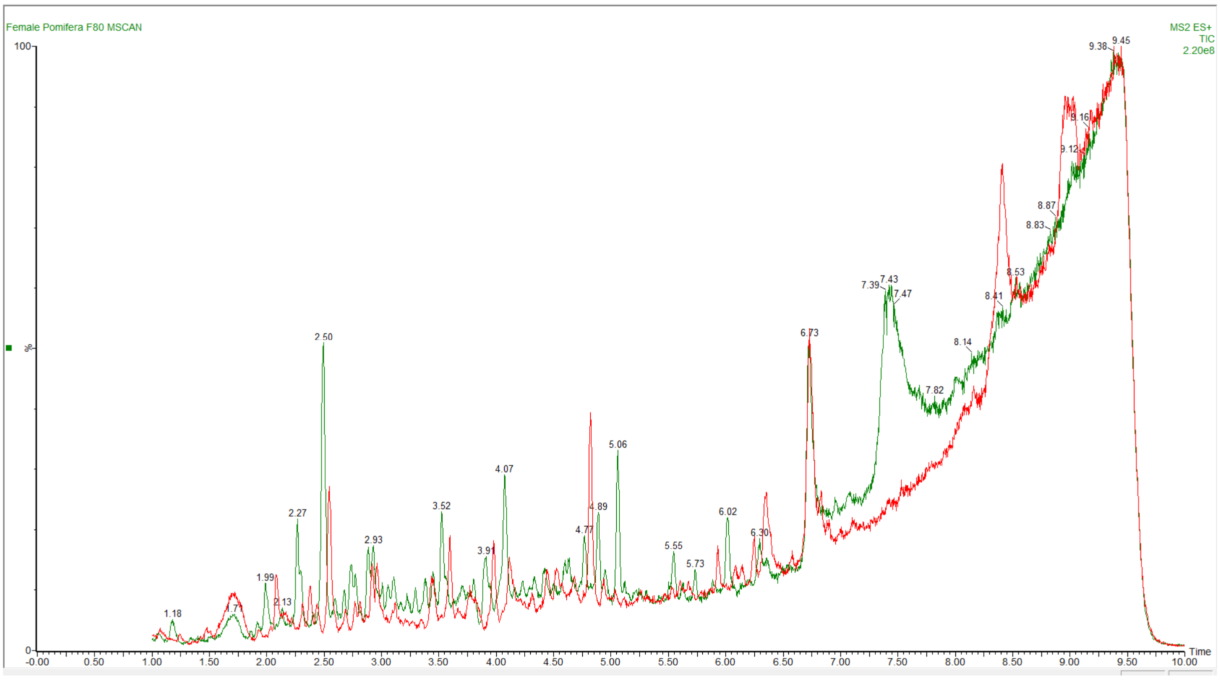

Figure S1: Representative UPLC profiles of *M. pomifera* male (red) and female (green) ethanol extracts in positive ionization mode.
